# Interstitial pneumonia in a glassblower: think to chronic beryllium disease!

**DOI:** 10.11604/pamj.2018.31.95.14831

**Published:** 2018-10-09

**Authors:** Amira Jamoussi, Takoua Merhabene, Mona Mlika, Henda Neji, Faouzi El Mezni, Jalila Ben Khelil, Mohamed Besbes

**Affiliations:** 1 Medical Intensive Care Unit, Abderrahmen Mami Hospital, Ariana 2080, Tunisia; 2 University of Tunis El Manar, Faculty of Medicine of Tunis, Tunis 1007, Tunisia; 3 Anatomopathology, Abderrahmen Mami hospital, Ariana 2080, Tunisia; 4 Medical Imaging, Abderrahmen Mami Hospital, Ariana 2080, Tunisia

**Keywords:** Lung toxicity, chronic beryllium disease, glassblower, interstitial pneumonia

## Abstract

Chronic beryllium disease (CBD) is an occupational illness with varying severity. In this report, we describe a 27 year old man, glassblower, who developed a fatal CBD after six months of unknown Beryllium's exposure. The diagnosis was suspected on histological examination and then consolidated by confirmation of Beryllium's exposure at the working area. Physicians should be aware of the potential risk to develop CBD in glassblowers. These workers should benefit from early medical surveillance using the Beryllium lymphocyte proliferation test (BeLPT) and therefore from suitable management.

## Introduction

Beryllium (Be) is a natural element used in several industries like automotive electronics, telecommunications, computers, aerospace and defense equipment, dental alloys and fluorescent lamps [1]. Workplace Be exposure can be unknown by employers, workers and even occupational physicians, thus leading to Chronic beryllium disease (CBD) misdiagnosis. We report a case of fatal CBD in a 27 years old man working in glass industry.

## Patient and observation

**Figure 1:**
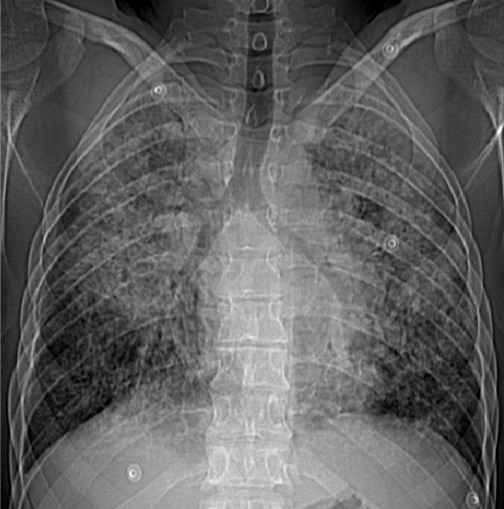
Chest X ray: bilateral alveolar opacities

**Figure 2:**
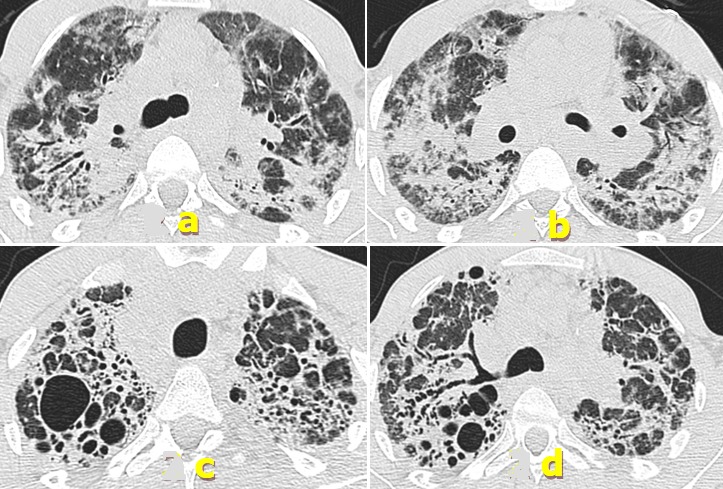
CT scan (lung windowing): a & b) bilateral lung consolidations and ground glass opacities with bronchial distortion; c & d) CT scan at 2 years: partial regression of lung consolidations and ground glass opacities and increase of distortion signs and cavities appearance

We report a 27 year old man, 10 pack-years smoker, with a one-month history of dry cough, shortness of breath and general status's deterioration. A lung X ray showed bilateral alveolar opacities (Figure 1). Blood gases analysis in ambient air showed a pO2 of 57 mmHg, a pCO2 of 38.6 mmHg, a HCO3- of 29.2 mmHg. Because of the considerable deterioration in his respiratory condition and the need for high oxygen flow, he was referred to intensive care unit (ICU) with the diagnosis of an acute respiratory failure (ARF). Chest computed tomography (CT) showed diffuse ground-glass opacities (Figure 2A, Figure 2B) and mediastinal lymphadenopathies. All microbiological infectious investigations, especially tuberculosis, were negative. He had hypergammaglobulinemia of 21.9 g/l. Other immunological tests were otherwise negative. Transthoracic echocardiography was performed finding a hyperkinetic left ventricle, a small dilation of right ventricle with PAPs 48 mmHg and an intrapulmonary shunt revealed by contrast test. The bronchial endoscopy showed an inflammatory bronchial tree and the bronchoalveolar lavage (BAL) concluded to lymphocytic alveolitis with a ratio CD4 (helper T cells) over CD8 (suppressor T cells) (CD4/CD8) of 0.5. The bronchial biopsies showed inflammatory bronchial mucosa without any specificities. Despite the absence of Koch's bacillus in sputum analysis, we decided to begin a trial anti-tuberculosis treatment because of the severity of lung involvement added to high endemicity of tuberculosis in our country. Two weeks later, the patient presented an allergic skin reaction. As no recovery's signs were noted, we decided to stop the treatment. Corticosteroids were initiated considering idiopathic diffuse infiltrating pneumonitis. Unfortunately, the patient did not show any improvement and the chest scan performed after 27 days of treatment showed worsening interstitial infiltrates and fibrosis.

**Figure 3:**
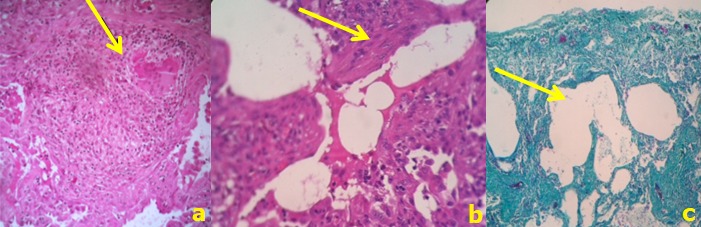
Microscopic exam: a) epitheloid granuloma; b) incorporation of hyaline membranes into the alveolar septa (arrow) (HE x 400); c) trichrom stain highlighting the honeycomb aspect (x 250)

Clinically, he required high flow oxygen (14 l/mn) alternating with non invasive ventilation sessions. Histological examination of the open lung's biopsy then performed showed diffuse lung parenchyma damage. It was altered by a mutilating fibrosis that was widening the alveolar septa. This fibrosis contained an abundant inflammatory infiltrate made of lymphocytes, plasmocytes and fewer neutrophils and eosinophils. Epithelioid and giant cell granulomas were also observed without either caseous or fibrinoïd necrosis. This mutilating fibrosis induced an enlargement of some alveoli resulting in the formation of a honeycomb aspect. Fibroblastic foci were also noticed. Some alveoli contained hyaline membranes that were sometimes incorporated in the septa. There were no mineral particles observed with polarized light (Figure 3). Regarding these features, the diagnosis of usual interstitial pneumonia (UIP) associated to lesions of sarcoidosis or berylliosis with aspects suggestive of an acute exacerbation was retained. Since sarcoidosis was not very likely according to CD4/CD8 ratio in BAL, we investigated risk factors for chronic beryllium disease, particularly occupation's patient history. He was working as glassblower in an artistic glass factory since six months. Further to our request, occupational department's experts inspected the workplace and confirmed beryllium existence in compounds of colored glass. After confirmation of CBD, steroids were maintained and oxygen requirement slowly decreased. The patient was finally discharged at home after 55 days steroid treatment with long term oxygen therapy (2 l/minute). Pulmonary function tests at discharge showed a severe restrictive impairement. Steroid doses were slowly decreased and stopped after 10 months. A pre-transplant assessment was also made. One year after discharge, the patient felt somewhat better and pulmonary function tests showed a slight improvement. The CT scan performed found a partial regression of lung consolidations and ground glass opacities, but increase of distortion signs, cavities appearance of which one filled by aspergilloma. He received oral voriconazole 200 mg per day during 3 months. After a two-year period from the date of CBD diagnosis, the patient was again admitted in ICU for ARF. An enhanced CT scan performed showed further deterioration of pulmonary parenchyma (Figure 2C, Figure 2D) and pulmonary embolism with fatal issue.

## Discussion

CBD is an occupational hypersensitivity disorder making a multisystem granulomatosis. It is caused by dust, fumes or mists of Be metal leading to lesions which occur primarily in the lungs [2]. Because of to the wide use of Be, exposure can occur in environments other than the traditional areas of Be manufacturing. As reported by the international agency for research on cancer (IARC) since 1993, additives to glass are considered as products with high potential Be exposure [2]. Among Be compounds, Beryllium-Oxide (BeO) is particularly used as an additive to glass, ceramics and plastics [3]. Thus, artistic glass industry is a potential situation to develop berylliosis. However, known lung diseases associated to glassblowing are pneumoconioses due to inhalation of inorganic dust like asbestos or silica [4, 5]. To our knowledge, this is the first case of CBD reported in a glassblower and we think that similar cases should exist but are misdiagnosed. Physicians must be aware of this risk and glassblowers should benefit from early screening using the beryllium lymphocyte proliferation test (BeLPT) [6] that confirms Be hypersensitivity preceding the disease. Indeed, at this stage, worker should be removed from his workplace, even if there is no evidence that Be exposure cessation firmly avoid progression of Be sensitization to CBD [7]. There are two clinical forms of Be related lung disease: an acute chemical pneumonitis due to a direct toxic injury of the lung, usually induced by brief exposure to high concentrations of Be, and completely reversible [8, 9] and CBD which is a multisystem immunologic granulomatosis which can resemble sarcoidosis or idiopathic diffuse interstitial fibrosis. It occurs after exposure to lower levels of Be with a long delay (several months to 20 years) between initial exposure and patent disease [8]. This observation highlights many particularities of clinical behavior: first, many factors leaded us to suspect the acute form of berylliosis: severity of initial clinical presentation, shortness of exposure delay, presence of hyaline membranes in some alveoli reminiscent of diffuse alveolar damage and direct exposure of the patient's respiratory tract. Indeed, glass-blowing offers special opportunities for exposure to metallic compounds in glass, which may migrate up the pipe into the worker's mouth and subsequently the bronchial tree [3]. Nevertheless, granulomas's presence at histological examination firmly established CBD diagnosis, and very short delays (4 months) had already been reported [8]. Second, we think that the first presentation was an acute exacerbation of usual interstitial pneumonia associated with CBD in a diffuse alveolar damage form at the fibrotic organization stage. Histological findings at the time of diagnosis of CBD have a prognosis value [8,10] which emphasize the importance of early medical surveillance using BeLPT [6].

## Conclusion

CBD diagnosis is usually based on unspecific findings. Furthermore, unsuspected Be exposure leads to misdiagnosis or to diagnosis and treatment delays. Glassblowers should undergo specific medical surveillance using the BeLPT to indicate their removal from Be exposure at the appropriate moment.

## Competing interests

The authors declare no competing interest.
